# The Relation Between the Excited Electronic States of Acene Radical Cations and Neutrals—A Computational Analysis

**DOI:** 10.1002/jcc.70095

**Published:** 2025-04-08

**Authors:** Anna M. Weidlich, Andreas Dreuw

**Affiliations:** ^1^ Interdisciplinary Center for Scientific Computing Ruprecht‐Karls University Heidelberg Germany

**Keywords:** acenes, ADC, excited states, open‐shell systems, radical cations

## Abstract

Acenes are a class of molecules that enjoy popularity in both experimental and theoretical fields of research for their diverse areas of application and unique electronic structure. One particular aspect of interest lies in their electronic absorption spectra, which have been thoroughly investigated both experimentally and theoretically. In this work, the electronically excited states of radical cations of acenes from naphthalene to dodecacene are investigated using algebraic diagrammatic construction (ADC) methods and different time‐dependent density functional theory (TD‐DFT) exchange‐correlation kernels. The performance of the employed ADC methods and different DFT functionals is assessed using experimental values as benchmarks. Using ADC, it is then shown that excited states typical for neutral acenes are retained in their radical cation counterparts, while additional states emerge due to excitations into the singly‐occupied molecular orbital (SOMO). Finally, the evolution of the excitation energies in neutral as well as cationic acenes with increasing length is investigated using TD‐DFT, where a special focus lies on the correct description of longer acenes using single‐reference methods.

## Graphical abstract

Neutral acenes represent an important molecular class for functional organic materials and organic electronics; however, their electronic structure is drastically changed upon ionization, forming radical cations. While the excited states of neutral acenes are well‐characterized, the corresponding radical cations have largely been overlooked. Here, a comprehensive computational investigation of their excited states, their relation to their neutral counterparts, and its behavior with increasing acene length is presented. The limits of their computational description are critically discussed.

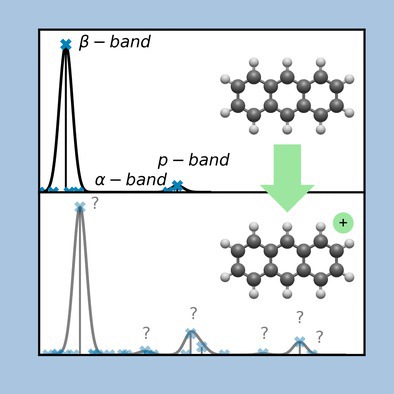

## 1 Introduction

Due to their unique electronic structure, acenes are an extensively studied class of molecules [1, 2, 3, 4, 5]. While there are numerous existing applications, a special interest lies in their potential use as organic semiconductors [6, 7, 8, 9]. Expanding their conjugated π‐system has a large impact on the electronic properties of acenes, such as the rapid decrease of the excitation energies of the electronic states. While this is a desirable property for their use as materials, it also causes a drastic decrease of their stability with respect to oxidation and dimerization, which is why acenes larger than hexacene are generally difficult to synthesize [4]. Furthermore, a decreasing S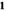 excitation energy is often related to an increasing diradical character of the electronic ground state. It has been shown that this is the case for acenes, which makes the correct theoretical description of longer acenes challenging [10, 11, 12].

Since the low‐energy excited states of acenes and their derivatives determine the suitability for different applications, they present a particular topic of interest. In this regard the excited states 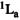, 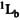, and 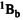, named after Platt's nomenclature, are the most relevant and are observed in the absorption spectra as the characteristic p‐, α‐ and β‐band respectively [1, 2]. The β‐band has the largest oscillator strength, while the p‐band shows a much weaker intensity and the α‐band almost none. In the molecular orbital picture, the 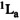 state (p‐band) is formed by a single‐electron H→L (H/HOMO—highest occupied molecular orbital, L/LUMO—lowest unoccupied molecular orbital) excitation. It has $B_{2u}$ symmetry and, except in case of naphthalene, is the energetically lowest‐lying transition. The 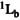 and 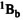 state (α‐ and β‐band) can be described by linear combinations of the transitions H‐1→L and H→L+1 (up to tetracene) and both belong to the irreducible representation $B_{3u}$.

Platt's nomenclature presents criteria for the naming of ππ* states in cata‐condensed hydrocarbons based on excitation energy, direction of polarization, oscillator strength and character of the transitions [1]. Identification of the 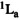 and 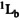 states based on these factors can however not be generally extended to derivatives of these systems such as *N*‐heteropolycycles [13]. Instead, the electron–hole correlation was identified to be a reliable physical criterion for their classification, and an alternative nomenclature of 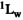 and 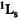, for weakly and strongly correlated was proposed. Accordingly, the 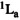 and 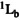 states have been renamed 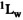 and 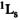 respectively. Furthermore, the distribution of the oscillator strength between $L_{s}$ and $B_{b}$ can be explained and influenced by the orbital energy difference of those orbitals participating in the contributing transitions [14]. In the example of anthracene, both the transitions H‐1→L and H→L+1 are mixed symmetrically, because they are energetically degenerate, leading to one state (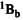) getting all the oscillator strength and the other one (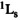) almost none. By derivatisation of the molecules, such as substitution with nitrogen, the energies of the participating molecular orbitals and therefore the distribution of the oscillator strength between the states can be adjusted [14].

Coming to radical cations with an unpaired α‐electron, new excitations occur involving transitions into the SOMO, in this case the β‐LUMO. Similar to neutral acenes, the excitation energy of the lowest excited electronic state decreases with increasing acene cation length, which impacts the theoretical description and leads to convergence issues (vide infra). While the excited states of neutral acenes have been extensively studied, much less is known about the excited states of their radical cations. Here, we present a thorough investigation of the excited states of acene cations and a comparison to the well‐known excited states of their neutral counterparts. We then explore the evolution of the excitation energies of the cations with increasing acene length. Ultimately, the fact that acene cations are open‐shell systems also allows for a fundamental investigation of how moving to the open‐shell picture impacts the electronic absorption spectra, the excited states as well as the employed methodology.

## 2 Methodology

All calculations were performed using the Q‐Chem 5.2 program package [15]. The investigated molecules are acenes of increasing length from naphthalene to dodecacene and their respective radical cations. For simplicity the acenes will be named according to the number of conjugated rings, for example, naphthalene is **2** and its cation is **2** 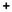. The geometries of all investigated molecules were obtained at the CAM‐B3LYP(D3(BJ))/6‐311G* level of theory [16, 17, 18]. The $\left\langle {\hat{S}}^{2}\right\rangle$‐values of **2** 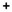 to **12** 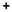 are 0.78, 0.78, 0.79, 0.79, 0.80, 0.80, 0.80, 0.81, 0.82, 0.83 and 0.84 respectively. The stationary points were confirmed to be minima by harmonic frequency analyses. Molecular symmetry of D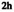 was ensured for all molecules. The basis set 6‐311G* was used in all excited state calculations [18].

To conform with existing literature, the molecules were oriented so that the x‐axis lies along the long molecular axis, the y‐axis along the short molecular axis and the z‐axis lies out of plane. This is also the orientation adopted by Q‐Chem, however it does not agree with the Mulliken convention. The term symbol of the ground state of acene cations with an even number of rings is A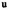, that of the acene cations with an uneven number of rings $B_{3g}$. In the benchmark of ADC methods against experiment, the low‐energy singlet/doublet excited states of **2**, **3** and **4** and their cations were calculated using ADC(2), ADC(2)‐x, ADC(3) and, only for the cations, IP‐ADC(3) [19, 20, 21, 22]. In case of IP‐ADC(3), excitation energies were obtained as energy differences between the lowest ionized state, that is, the cationic ground state, and the respective higher‐lying ionized states. The obtained vertical excitation energies are compared to experimental absorption spectra. Although vibrational contributions may be relevant for a quantitative agreement, they are in the order of 0.05 eV for vertical excitation energies and unlikely to significantly influence the outcome of our study [23, 24].

The experimental excitation energies of acene cations were taken from literature, where the ions were created using vacuum ultraviolet irradiation or vapor phase electron impact prior to the absorption spectra being measured in argon and/or neon matrices [25, 26, 27]. The benchmark of DFT functionals against experimental values comprises the low‐energy singlet/doublet excited states of **2**, **3**, **4** and **5** and their cations calculated using BLYP, B3LYP, BHHLYP and CAM‐B3LYP at full TD‐DFT level of theory without employing the Tamm‐Dancoff approximation (TDA) [16, 28, 29, 30, 31, 32]. For comparison the excited states of **2** 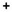
–**12** 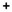 and **2**–**12** are calculated at the TDA/CAM‐B3LYP/6‐311G* level of theory. It was not possible to perform a full TD‐DFT calculation for acenes larger than nonacene, which is most likely due to triplet instabilities. For acenes up to **9**, the triplet ground state is higher in energy than the closed‐shell singlet ground state. For **10**–**12**, the triplet becomes lower in energy. This can be explained by the increasing diradical character and inability of (TD)‐DFT to describe an open‐shell singlet ground state. It has been shown that the multiplicity of the ground state of acenes is a singlet for all acene lengths [11]. In the excited state calculations of acene cations larger than **9** 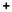, orbital degeneracies cause the SCF to converge to an unstable solution resulting in a negative excitation energy for the first excited state, which is described by the transition αH→αL. In order to obtain only positive excitation energies, the αH and αL are switched in the initial guess. This lead to a lower final SCF energy. For the assignment of neutral and cationic orbitals in the analysis of the ADC excited states, the HF orbitals were visualized using IQmol 2.8.0. The absorption spectra were simulated by convolution of the excitation energies using a Gaussian broadening function with a standard deviation of 0.1 eV.

## 3 ADC Benchmark and Characterization of Excited States of Naphthalene, Anthracene, Tetracene, and Their Radical Cations

### 3.1 Naphthalene

As mentioned above, all neutral acenes display typical absorption spectra containing the p‐, α‐ and β‐band, where the corresponding excited states 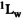, 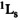, and 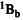 have characteristic irreducible representations and orbital contributions to their main transitions. The calculated excited states of **2** using ADC(2), ADC(2)‐x and ADC(3) are assigned according to their irreducible representations and orbital transitions. The respective excitation energies are compared to experimental values. While overall the ADC methods overestimate the excitation energies of **2**, Table 1 shows that ADC(2)‐x gives the best accordance with experimental values. It has been observed that ADC(2)‐x consistently underestimates excitation energies, which in this case makes them match the experimental values fortuitously more closely [33].

**TABLE 1:** Excitation energies (eV) and main orbital contributions of , and of **2**, calculated using ADC(2), ADC(2)‐x and ADC(3), as well as experimental values.

State	Exp [3]	ADC(2)	ADC(2)‐x	ADC(3)	Main contrib.
^1^L_w_ (1^1^B_2u_, p‐band)	4.38	5.06	4.58	5.04	H → L
^1^L_s_ (1^1^B_3u_, *α*‐band)	4.03	4.6	3.62	4.29	H‐1 → L
					H → L + 1
^1^B_b_ (2^1^B_3u_, *β*‐band)	5.62	6.39	5.75	6.44	H → L + 1
					H‐1 → L
MAE (eV)		0.67	0.25	0.58	
MAPE (%)		12.6	6.0	10.6	

The low‐energy excited states of **2** 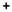 were calculated using ADC(2), ADC(2)‐x, ADC(3) and IP‐ADC(3) and compared to experimental values. The excited state assignment is based on their excitation energies and irreducible representations. In best agreement with experimental values are ADC(3) and IP‐ADC(3), which show small mean absolute errors (MAEs) and mean absolute percentage errors (MAPEs) of only 0.18 eV (5.3%) and 0.23 eV (7.0%) respectively (Table 2).

**TABLE 2:** Excitation energies (eV) of **2**, calculated using ADC(2), ADC(2)‐x, ADC(3), IP‐ADC(3), as well as experimental values.

State [25, 34]	Exp [25]	ADC(2)	ADC(2)‐x	ADC(3)	IP‐ADC(3)
1^2^B_1u_	forb.	1.36	0.49	1.03	0.66
1^2^B_3g_	1.83	2.08	1.31	1.82	2.08
1^2^B_2g_	2.72	3.26	2.29	2.94	3.03
2^2^B_2g_	3.29	3.84	2.87	3.47	3.28
2^2^B_3g_	4.02	4.24	3.23	3.73	4.28
3^2^B_3g_	4.49	4.92	3.66	—	4.8
3^2^B_2g_	5.08	5.27	4.31	4.86	5.57
4^2^B_2g_	5.58	6.91	5.41	5.66	6.24
MAE (eV)		0.40	0.60	0.18	0.23
MAPE (%)		11.4	24.0	5.3	7.0

**FIGURE 1:**
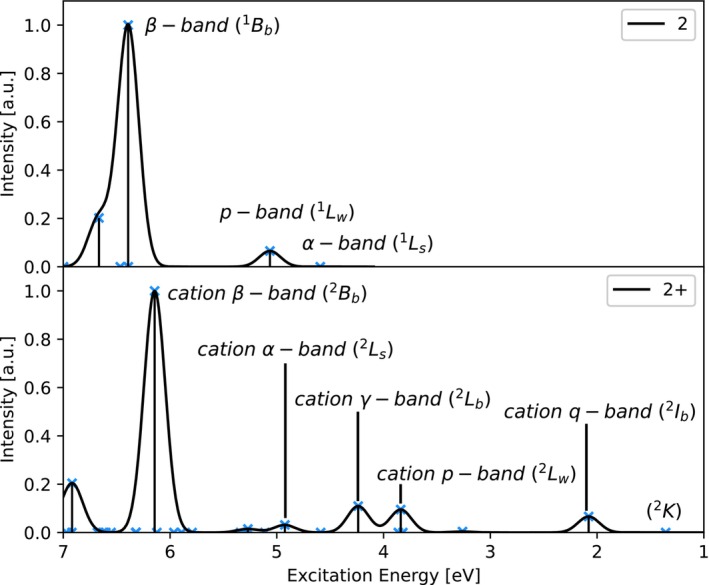
Simulated absorption spectra of **2** (top) and **2** 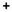 (bottom) obtained by convolution of excitation energies (ADC(2)/6‐311G*) using a Gaussian broadening function with a standard deviation of 0.1 eV.

The low‐energy excitations of **2** 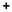 are now compared with and, if possible, assigned to the typical excited states of the neutral acene **2** by means of their prominent orbital contributions. To do that the orbitals of **2** 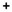 need to be assigned to those of **2** according to their shape and symmetry. An example for the assignment of relevant orbitals can be found in Figure S1 and Table S1 in the Supporting Information. Using this assignment, the excited states of **2** 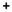 that are described by the same orbital transition as the 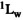, 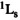 and 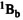 states of its neutral counterpart **2** can be identified (Table S1). Since in the open‐shell picture α‐ and β‐orbitals are not equivalent, each orbital of a neutral acene has one α‐ and one β‐counterpart in the cation. Therefore also each transition has two counterparts in the cationic acenes for example, the H‐1→L transition in **2** corresponds to both the αH→αL transition as well as the βH‐1 →βL+1 transition (see Table S1). It shall be noted here that ADC(2) results were used as a basis for the assignment because, compared to ADC(2)‐x and ADC(3), double excitations are included in zeroth order only, making state relation of excited states of cationic and neutral species unambiguous and thereby facilitating the comparison with the subsequent TD‐DFT calculations. It can be seen that the characteristic acene excited states are still present in the cation, while naturally new states appear due to the excitations involving the β‐LUMO, which is otherwise occupied in the neutral compound. This is reflected in the absorption spectra shown in Figure 1. The lowest excited state, 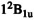, which is primarily described by the transition βH‐1 →βL, has nearly no oscillator strength and obviously no counterpart in the neutral compound. Platt's nomenclature labels states according to the orientation of the transition dipole moment relative to the long molecular axis (perpendicular (a) or parallel (b)) and the number of nodal planes on the perimeter of the molecule of the excited states polarization diagram. States with 2n, 2n+1 and 2n+2 nodal planes, where n is the number of aromatic rings, are labeled K, L, and M. Following Platt's nomenclature, this state is labeled 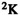. The next excitation, 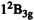, is mainly described by βH→βL and is the first state with considerable oscillator strength. This state is labeled 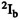, since the number of nodal planes relates to the number of aromatic rings with 2n‐2. The corresponding absorption band will be called cation q‐band in reference to the p‐band in neutral acenes. The following excited state, 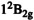, is described by two transitions, αH‐1 →αL and βH‐2→βL. According to the orbital assignment, the transition αH‐1 →αL in the cation corresponds to the H→L transition in the neutral compound. The state described by this transition is therefore assigned to the 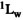 state (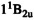, p‐band) in the neutral compound. However in this case, due to the mixing with the transition βH‐2→βL, this state is split up into two states. The other one is 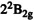. Here the second state can be identified as the 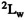 state by comparing the transition density to that of the neutral 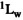 and the corresponding absorption band will be called the cation p‐band. As 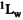 in **2**, this state shows an intermediate intensity. It can further be seen that both the 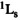 state (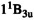, α‐band) and the 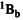 state (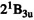, β‐band) of **2** have counterparts in the cationic compound, after assignment of the orbitals, 3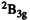 and 4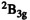, which will be labeled 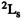 and 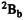. The absorption bands of the cationic counterparts will be called cation α‐band and cation β‐band in the following. Similar to the neutral acenes, the cation β‐band features the highest oscillator strength, while the cation α‐band is nearly completely dark.

When looking at the excited states, it is apparent that there is a third state, 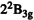, which is described by the two contributions that both correspond to H‐1→L. Following Platt's nomenclature, this state is denoted as 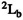. However, a large $\left\langle {\hat{S}}^{2}\right\rangle$‐values of 2.5 indicates considerable spin‐contamination arising from insufficient inclusion of doubly excited configurations that, in open‐shell systems, would be needed for a spin‐complete description of the transition. The results for this state thus need to be considered carefully. It should however be noted that the IP‐ADC(3) description does not suffer from this issue, because it builds upon a closed‐shell reference state (the neutral acene). Comparing the excitation energies from ADC(2) and IP‐ADC(3), it can be seen that they are similar with 4.24 and 4.28 eV respectively. The related absorption band will be called cation γ‐band in the following as a continuation of the α‐ and β‐band nomenclature.

It is important to note that in neutral acenes the irreducible representations of the 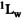, 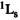 and 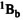 excited states, 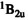 and 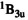, do not change between acenes with different numbers of rings. Meanwhile the ground states of cationic acenes have a different irreducible representation depending on the number of rings being even or odd and therefore also the excited states of the same character have different irreducible representations for even and odd numbers of rings. In the following, labels for states and their absorption bands will therefore be given in accordance with the assignment in Table 3 depending on the orbital contributions.

**TABLE 3:** Excitation energies (eV), oscillator strengths, orbital contributions and assignment of ADC(2) states of **2**.

ADC(2)	*f*_osc_ [10^−2^]	Main contrib.	Assignment neutral	State (name)
1.36	0.00	*β*H‐1 → *β*L	—	1^2^B_1u_ (^2^K)
2.08	0.73	*β*H → *β*L	—	1^2^B_3g_ (^2^I_b_, cation q‐band)
3.26	0.04	*β*H‐2 → *β*L	—	1^2^B_2g_
		*α*H‐1 → *α*L	H → L	
3.84	1.05	*α*H‐1 → *α*L	H → L	2^2^B_2g_ (^2^L_w_, cation p‐band)
		*β*H‐2 → *β*L	—	
4.24	1.21	*α*H → *α*L	H‐1 → L	2^2^B_3g_ (^2^L_b_, cation *γ*‐band)
		*β*H‐1 → *β*L + 1	H‐1 → L	
4.92	0.35	*β*H‐1 → *β*L + 1	H‐1 → L	3^2^B_3g_ (^2^L_s_, cation *α*‐band)
		*α*H‐1 → *α*L + 1	H → L + 1	
6.14	11.07	*α*H‐1 → *α*L + 1	H → L + 1	4^2^B_3g_ (^2^B_b_, cation *β*‐band)
		*β*H‐1 → *β*L + 1	H‐1 → L	

Simulated absorption spectra of **2** and **2** 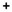 can be seen in Figure 1, where the states of interest are labeled. Comparing the cationic absorption bands to their neutral counterparts in Figure 1, it is easily noticed that the cation α‐band at 4.92 eV is slightly blue‐shifted, while the cation β‐band at 6.14 eV is slightly red‐shifted compared to 4.60 eV and 6.39 eV respectively in the neutral compound. Also the cation α‐band gains some oscillator strength as compared to the neutral α‐band. The cation p‐band appears at a lower energy at 3.84 eV than the neutral p‐band at 5.06 eV, most likely due to mixing with the βH‐2 → βL transition. The simulated absorption spectra obtained using ADC(2)‐x, ADC(3) and IP‐ADC(3) excitation energies can be found in Figures S2 to S4.

### 3.2 Anthracene

The corresponding analysis is done for **3**. The calculated excitation energies given by ADC(2), ADC(2)‐x and ADC(3) for 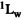, 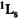 and 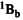 are compared to the experimental excitation energies of the p‐, α‐ and β‐band. The results can be found in Table 4. As before, the closest agreement with experimental values is achieved with ADC(2)‐x.

**TABLE 4:** Excitation energies (eV) and main orbital contributions of , and of **3**, calculated using ADC(2), ADC(2)‐x and ADC(3), as well as experimental values.

State	Exp [3]	ADC(2)	ADC(2)‐x	ADC(3)	Main contrib.
^1^L_w_ (1^1^B_2u_, p‐band)	3.38	3.88	3.40	3.89	H → L
^1^L_s_ (1^1^B_3u_, *α*‐band)	3.57	4.02	3.11	3.72	H‐1 → L
					H → L + 1
^1^B_b_ (2^1^B_3u_, *β*‐band)	4.86	5.59	5.10	5.66	H → L + 1
					H‐1 → L
MAE (eV)		0.56	0.24	0.49	
MAPE (%)		12.4	6.7	10.4	

Subsequently, also the low‐energy excited states of **3** 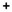 were calculated using ADC(2), ADC(2)‐x, ADC(3) and IP‐ADC(3) and compared to experimental values in Table 5. The assignment to experimental states is based on excitation energies and symmetry. Again ADC(3) and IP‐ADC(3) show very good agreement with experiment with an MAE (MAPE) of only 0.19 eV (7.3%) and 0.21 eV (8.4%) respectively (Table 6).

**TABLE 5:** Excitation energies (eV) of **3**, calculated using ADC(2), ADC(2)‐x, ADC(3), IP‐ADC(3), as well as experimental values.

State [26]	Exp [26]	ADC(2)	ADC(2)‐x	ADC(3)	IP‐ADC(3)
1^2^B_2g_	forb.	1.81	0.93	1.44	1.14
1^2^A_u_	1.73	2.00	1.20	1.76	1.94
1^2^B_1u_	2.02	2.56	1.76	2.29	2.11
1^2^B_3g_	forb.	—	2.30	2.92	—
2^2^B_1u_	2.90	3.50	2.51	3.17	3.23
3^2^B_1u_	3.52	—	—	—	—
2^2^A_u_	3.95	3.67	2.82	3.28	3.84
MAE (eV)		0.47	0.39	0.19	0.21
MAPE (%)		17.2	24.8	7.3	8.4

*Note:* A different molecular orientation was used in [26] resulting in a different labeling of some states: $B_{3g}$ in [26] corresponds to $B_{2g}$ in this work, and vice versa.

**TABLE 6:** Excitation energies (eV), oscillator strengths, orbital contributions and assignment of ADC(2) states of **3**.

ADC(2)	*f*_osc_ [10^−2^]	Main contrib.	Assignment neutral	State (name)
1.81	0.00	*β*H‐1 → *β*L	—	1^2^B_2g_ (^2^K)
2.00	1.43	*β*H → *β*L	—	1^2^A_u_ (^2^I_b_, cation q‐band)
2.56	0.15	*α*H → *α*L	H → L	1^2^B_1u_ (^2^L_w_, cation p‐band)
3.67	2.34	*α*H‐1 → *α*L	H‐1 → L	2^2^A_u_ (^2^L_b_, cation *γ*‐band)
		*β*H‐1 → *β*L + 1	H‐1 → L	
4.37	0.42	*β*H‐1 → *β*L + 1	H‐1 → L	3^2^A_u_ (2*L*_s_, cation *α*‐band)
		*α*H → *α*L + 1	H → L + 1	
5.37	16.34	*α*H → *α*L + 1	H → L + 1	4^2^A_u_ (^2^B_b_, cation *β*‐band)
		*β*H‐1 → *β*L + 1	H‐1 → L	

**FIGURE 2:**
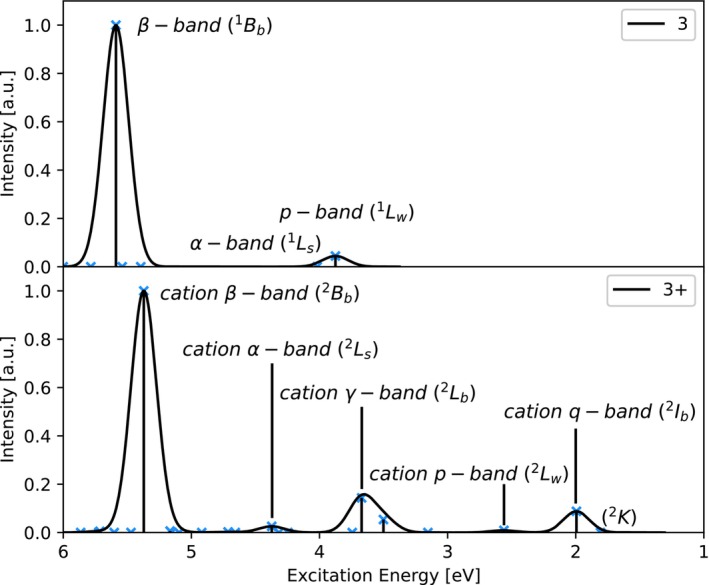
Simulated absorption spectra of **3** (top) and **3** 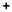 (bottom) obtained by convolution of excitation energies (ADC(2)/6‐311G*) using a Gaussian broadening function with a standard deviation of 0.1 eV.

By assignment of the relevant orbitals of **3** to those of **3** 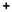 in Table S2, the states that correspond to the typical acene states can be identified. As is evident from Figure 2, a similar picture as for **2** 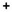 is found also for **3** 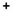. The first excited state, 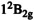, is described mainly by the transition βH‐1 →βL and has nearly no oscillator strength. It therefore corresponds to the 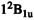 state in **2** 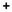 and is labeled 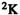. The following excited state, 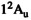, can be described by the transition βH→βL and therefore corresponds to the excited state 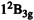 in **2** 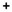, which is labeled 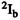 (cation q‐band). As mentioned before, both of these states do not have counterparts in the neutral molecule. The next excited state, 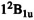, can however be assigned to the 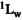 state in neutral anthracene based on orbital contributions and therefore corresponds to 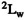 and the absorption band to the cation p‐band. An assignment of the 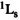 and 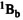 states (α‐ and β‐band) in neutral anthracene can also be made to the excited states 3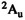 and 4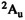 (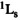, cation α‐band and 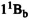, cation β‐band).

Simulated absorption spectra of **3** and **3** 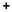 can be seen in Figure 2, where the states of interest are labeled. It can be seen that the cation α‐ and β‐bands are similar in appearance to the neutral counterparts. As for **2** 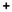, a smaller energy difference between the cation α‐ and β‐state at 4.37 eV and 5.37 eV respectively is observed in **3** 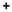 as compared to those of **3** at 4.02 eV and 5.59 eV. Also the cation α‐band features an enhanced absorption intensity as compared to its neutral counterpart. Different from **2** 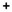, the cation p‐band at 2.56 eV is not mixed with other transitions and now shows an excitation energy closer to the neutral p‐band at 3.88 eV. The simulated absorption spectra obtained using ADC(2)‐x, ADC(3) and IP‐ADC(3) excitation energies can be found in Figures S5 to S7.

### 3.3 Tetracene

Comparing the 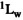, 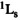 and 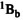 states of **4** calculated at ADC levels of theory with experimental values for the p‐band, α‐band and β‐band, ADC(3) achieves the best agreement, which can be seen in Table 7.

**TABLE 7:** Excitation energies (eV) and main orbital contributions of , and of **4**, calculated using ADC(2), ADC(2)‐x and ADC(3), as well as experimental values.

State	Exp [3]	ADC(2)	ADC(2)‐x	ADC(3)	Main contrib.
^1^L_w_ (1^1^B_2u_, p‐band)	2.71	3.08	2.52	3.10	H → L
^1^L_s_ (1^1^B_3u_, *α*‐band)	3.32	3.65	2.75	3.34	H‐1 → L
					H → L + 1
^1^B_b_ (2^1^B_3u_, *β*‐band)	4.52	5.03	4.45	4.34	H → L + 1
					H‐1 → L
MAE (eV)		0.40	0.28	0.20	
MAPE (%)		10.4	10.0	5.8	

The low‐energy excited states of **4** 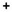 calculated using ADC(2), ADC(2)‐x, ADC(3) and IP‐ADC(3) are shown in Table 8 and also compared to experimental values. As before, the assignment of the states is based on excitation energies and irreducible representations. ADC(3) and IP‐ADC(3) again show by far the best agreement with an MAE (MAPE) of 0.11 eV (4.9%) and 0.05 eV (2.5%) respectively.

**TABLE 8:** Excitation energies (eV) of **4**, calculated using ADC(2), ADC(2)‐x, ADC(3), IP‐ADC(3), as well as experimental values.

State [27]	Exp [27]	ADC(2)	ADC(2)‐x	ADC(3)	IP‐ADC(3)
1^2^B_3g_	1.43	1.77	1.00	1.52	1.44
1^2^B_1u_	forb.	2.15	1.27	1.76	1.49
(1^2^B_2g_)	1.66	1.95	1.21	1.68	1.73
1^2^A_u_	forb.	3.09	2.23	2.88	3.03
2^2^B_2g_	—	3.44	2.49	3.10	3.48
2^2^B_3g_	3.16	3.27	2.53	2.94	3.17
MAE (eV)		0.25	0.50	0.11	0.05
MAPE (%)		12.5	35.0	4.9	2.5

*Note:* A different molecular orientation was used in [27] resulting in a different labeling of some states: $B_{3g}$ in [27] corresponds to $B_{2g}$ in this work, and vice versa.

The excited states of **4** 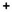 are then assigned according to the same procedure outlined above (Table 9). The assignment of the relevant orbitals can be found in Table S3. Different from **2** 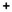 and **3** 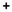, the lowest‐lying excited state, 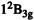, is described by the transition βH→βL and therefore corresponds to the excited state 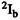. The following excited state 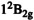 corresponds to the 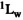 (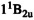) state in neutral tetracene and is therefore labeled 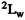. After that, we can see the excited state 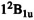 that has no oscillator strength and can be described by the transition βH‐1 →βL that is labeled 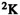. The states that can be assigned to the 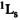 and 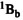 states in neutral tetracene are found in the cation as 4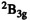 and 5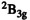 (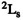, cation α‐ and 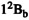, cation β‐band).

**TABLE 9:** Excitation energies (eV), oscillator strenghts, orbital contributions and assignment of ADC(2) states of **4**.

ADC(2)	OSC strength	Main contrib.	Assignment neutral	State (name)
1.77	2.15	*β*H → *β*L	—	1^2^B_3g_ (^2^I_b_, cation q‐band)
1.95	0.22	*α*H → *α*L	H → L	1^2^B_2g_ (^2^L_w_, cation p‐band)
2.15	0.00	*β*H‐1 → *β*L	—	1^2^B_1u_ (^2^K)
3.27	4.03	*α*H‐1 → *α*L	H‐1 → L	2^2^B_3g_ (^2^L_b_, cation *γ*‐band)
		*β*H‐1 → *β*L + 1	H‐1 → L	
4.04	0.58	*β*H‐1 → *β*L + 1	H‐1 → L	4^2^B_3g_ (^2^L_s_, cation *α*‐band)
		*α*H → *α*L + 2	H → L + 1	
4.88	21.09	*α*H → *α*L + 2	H → L + 1	5^2^B_3g_ (^2^B_b_, cation *β*‐band)
		*β*H‐1 → *β*L + 1	H‐1 → L	

**FIGURE 3:**
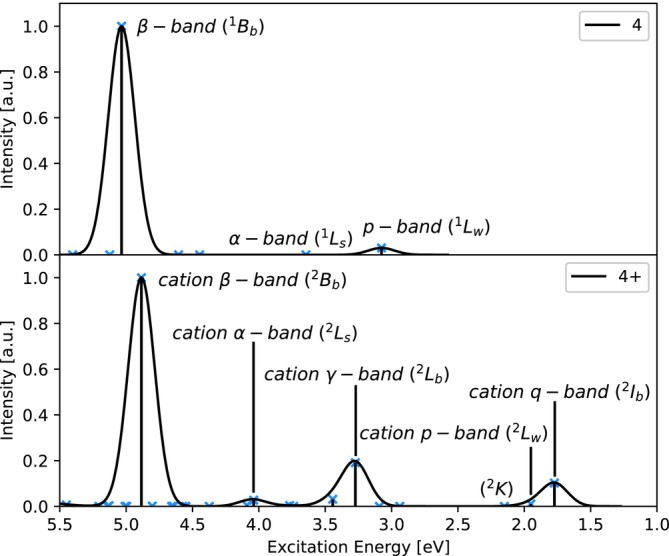
Simulated absorption spectra of **4** (top) and **4** 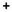 (bottom) obtained by convolution of excitation energies (ADC(2)/6‐311G*) using a Gaussian broadening function with a standard deviation of 0.1 eV.

Simulated absorption spectra of **4** and **4** 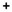 can be seen in Figure 3, where the states of interest are labeled. As before, the cation β‐band is slightly red‐shifted, while the cation α‐band is slightly blue‐shifted as compared to their neutral counterparts and the cation α‐band gains some oscillator strength. The associated excitation energies are 3.65 eV and 5.03 eV for the states of the neutral molecule and 4.04 eV and 4.88 eV for the cationic ones. The excitation energies of the neutral p‐band and cation p‐band are 3.08 eV and 1.95 eV respectively and therefore show a slightly smaller energy difference than in **3** 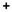. The simulated absorption spectra obtained using ADC(2)‐x, ADC(3) and IP‐ADC(3) excitation energies can be found in Figures S8 to S10.

## 4 TD‐DFT Benchmark of Excited States of Naphthalene, Anthracene, Tetracene, Pentacene, and Their Radical Cations

The low‐energy electronic excited states of **2**–**5** and their respective cations are calculated at the full TD‐DFT level using the functionals BLYP, B3LYP, BHHLYP, and CAM‐B3LYP. The states were assigned according to their orbital contributions and excitation energies and compared to experimental values. The results are shown in Tables 10, 11, 12, 13.

**TABLE 10:** Excitation energies (eV) of low‐lying excited states of **2** and **2** calculated using the BLYP, B3LYP, BHHLYP and CAM‐B3LYP functionals and comparison to experimental values.

State [3, 25, 34]	Exp [3, 25]	BLYP	B3LYP	BHHLYP	CAM‐B3LYP
Neutral					
1^1^B_2u_	4.48	4.17	4.48	4.80	4.79
1^1^B_3u_	4.03	4.31	4.56	4.84	4.72
2^1^B_3u_	5.62	5.88	6.10	6.39	6.28
MAE (eV)		0.25	0.37	0.67	0.59
MAPE (%)		5.3	7.2	12.5	11.2
Cation					
1^2^B_1u_	forb.	1.08	1.17	1.31	1.22
1^2^B_3g_	1.83	2.19	2.19	2.14	2.14
1^2^B_2g_	2.72	2.84	3.04	3.24	3.28
2^2^B_2g_	3.29	3.56	3.65	3.46	3.65
2^2^B_3g_	4.02	3.80	3.97	4.09	4.06
3^2^B_3g_	4.49	4.35	4.66	5.12	4.94
3^2^B_2g_	5.08	4.60	4.66	5.12	4.94
MAE (eV)		0.19	0.21	0.28	0.31
MAPE (%)		7.9	7.9	12.3	9.1

**TABLE 11:** Excitation energies (eV) of low‐lying excited states of **3** and **3** calculated using the BLYP, B3LYP, BHHLYP and CAM‐B3LYP functionals and comparison to experimental values.

State [3, 26]	Exp [3, 26]	BLYP	B3LYP	BHHLYP	CAM‐B3LYP
Neutral					
1^1^B_2u_	3.38	3.01	3.13	3.67	3.67
1^1^B_3u_	3.57	3.69	3.95	4.23	4.13
2^1^B_3u_	4.86	5.06	5.33	5.64	5.55
MAE (eV)		0.23	0.31	0.58	0.51
MAPE (%)		6.5	6.8	12.4	11.2
Cation					
1^2^B_2g_	forb.	1.45	1.57	1.74	1.66
1^2^A_u_	1.73	1.90	1.97	2.08	2.03
1^2^B_1u_	2.02	2.24	2.36	2.35	2.48
1^2^B_3g_	forb.	2.84	3.03	3.27	3.26
2^2^B_1u_	2.90	2.96	3.22	3.35	3.42
MAE (eV)		0.15	0.30	0.38	0.43
MAPE (%)		6.9	12.2	14.8	16.3

*Note:* The molecular orientation in reference [26] differs from the one in this paper so that $B_{3g}$ in the reference corresponds to $B_{2g}$ here.

**TABLE 12:** Excitation energies (eV) of low‐lying excited states of **4** and **4** calculated using the BLYP, B3LYP, BHHLYP and CAM‐B3LYP functionals and comparison to experimental values.

State [3, 27]	Exp [3, 27]	BLYP	B3LYP	BHHLYP	CAM‐B3LYP
Neutral					
1^1^B_2u_	2.71	2.24	2.54	2.88	2.90
1^1^B_3u_	3.32	3.29	3.56	3.83	3.75
2^1^B_3u_	4.52	4.48	4.77	5.11	5.03
MAE (eV)		0.18	0.22	0.42	0.38
MAPE (%)		7.6	6.3	10.2	9.4
Cation					
1^2^B_3g_	1.43	1.64	1.73	1.67	1.83
1^2^B_1u_	forb.	1.68	1.84	1.95	1.94
(1^2^B_2g_)	1.66	1.65	1.74	1.85	1.80
1^2^A_u_	forb.	2.53	2.80	3.20	3.17
2^2^B_2g_	—	2.91	3.43	3.18	3.19
2^2^B_3g_	3.16	2.77	3.08	3.20	3.32
MAE (eV)		0.20	0.15	0.20	0.23
MAPE (%)		9.2	8.2	9.9	11.5

*Note:* The molecular orientation in reference [26] differs from the one in this paper so that $B_{3g}$ in the reference corresponds to $B_{2g}$ here.

**TABLE 13:** Excitation energies (eV) of low‐lying excited states of **5** and **5** calculated using the BLYP, B3LYP, BHHLYP and CAM‐B3LYP functionals and comparison to experimental values.

State [3, 35]	Exp [3, 35]	BLYP	B3LYP	BHHLYP	CAM‐B3LYP
Neutral					
1^1^B_2u_	2.23	1.70	1.99	2.35	2.31
1^1^B_3u_	3.05	3.01	3.28	3.49	3.55
2^1^B_3u_	4.14	4.05	4.37	4.65	4.71
MAE (eV)		0.22	0.23	0.36	0.38
MAPE (%)		11.6	8.1	9.6	9.9
Cation					
1^2^B_1u_	1.27	1.19	1.27	1.15	1.34
1^2^A_u_	1.31	1.46	1.54	1.66	1.61
2^2^A_u_	2.95	2.65	2.84	2.92	2.94
MAE (eV)		0.13	0.09	0.13	0.09
MAPE (%)		9.4	6.3	10.8	8.0

For the neutral acenes, the best agreement with experimental values for the excitation energies of the p‐, α‐ and β‐band overall is obtained for the BLYP and B3LYP functionals. However, the agreement of BLYP seems to decline as we move to longer acenes with MAPEs of 5.3%, 6.5%, 7.6%, and 11.6% for **2** to **5**. For the B3LYP functional, the MAPEs are more constant for all neutral acenes with values between 6.3% and 8.1%. The agreement of BHHLYP and CAM‐B3LYP on the other hand seems to improve as the length of the acene is increased with MAPE of 12.5%, 12.4%, 10.2%, and 9.6% and 11.2%, 11.2%, 9.4%, and 9.9% for **2**–**5** respectively.

In case of the acene cations, BLYP shows very good agreement with experiment with MAPEs of 7.9%, 6.9%, 9.2%, and 9.4% for **2** 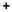
‐**5** 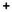, while as for the neutral acenes, the performance seems to decline with increasing acene length. The B3LYP functional also shows good agreement with experimental values with MAPEs of 7.9%, 12.2%, 8.2%, and 6.3% for **2** 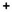
‐**5** 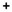. This is in accordance with previous studies of the performance of the functionals BLYP and B3LYP for small cationic acenes [36]. For the shorter acenes, the functionals BHHLYP and CAM‐B3LYP perform worse with MAPEs of 12.3% and 14.8% as well as 9.1% and 16.3% for **2** 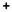 and **3** 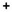 respectively. For the longer acenes **4** 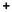 and **5** 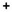 the MAPEs are improved with 9.9% and 10.8% for BHHLYP and 11.5% and 8.0% for CAM‐B3LYP. Considering the overall performance of the evaluated functionals, one notices the trend that the longer the acene gets, the smaller the difference in MAPEs becomes, with the respective values for BLYP, B3LYP, BHHLYP and CAM‐B3LYP being 9.4%, 6.3%, 10.8% and 8.0% for **5** 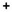 respectively. It has been observed that for the correct description of excited states in extended π‐system using TD‐DFT, the use of a long‐range separated functional is necessary [37, 38, 39]. This is especially important considering prospective investigations of exciton properties, which are strongly dependent on the inclusion of a long‐range correction [40, 41]. For these reasons and considering that the performance of the different functionals is similar for larger acenes, the CAM‐B3LYP functional is chosen for further calculations.

## 5 Excited States of Naphthalene to Dodecacene and Their Radical Cations

**FIGURE 4:**
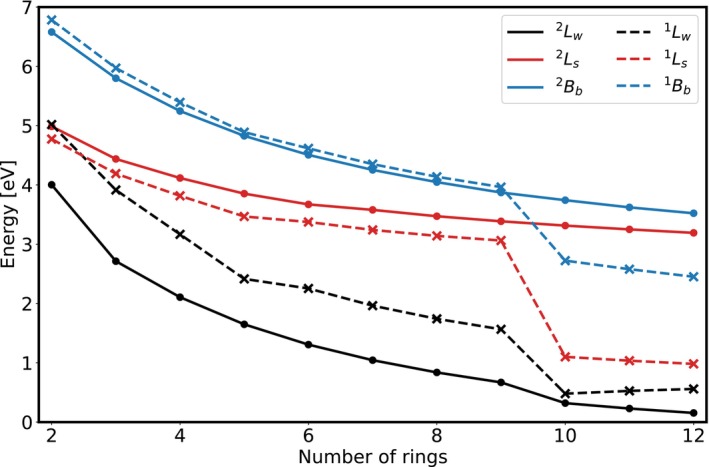
Excitation energies of selected excited states of **2** 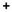
‐**12** 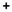 (solid lines) and **2**–**12** (dotted lines) at TDA/CAM‐B3LYP/6‐311G*.

Electronically excited states of **2** 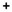
‐**12** 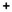 and for comparison **2**–**12** are calculated at the TDA/CAM‐B3LYP/6‐311G* level of theory and assigned according to their orbital contributions. The trend in the excitation energies of the 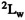, 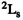 and 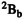 states of **2** 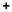
‐**12** 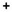 as well as 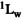, 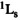 and 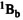 of **2**–**12** can be seen in Figure 4. The corresponding simulated excitation spectra can be found in the Supporting Information (Figures S11 and S12).

All selected excited states show the expected red‐shift with increasing acene length. Furthermore all excitation energies seem to be converging towards a certain value as the acenes get longer. For both the neutral and the cationic acenes, the order of the shown states does not change (except for 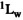 and 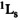 of **2**) with the state 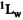/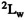 being lowest in energy followed by 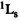/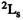 and 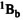/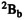 respectively. The energy difference between the 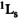/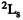 and 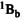/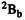 states decreases as the acenes get longer. It can be seen for 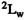 that there is a slight discontinuity when going from **9** 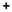 to **10** 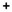, while the 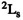 and 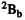 state do not seem to be affected. In case of the neutral acenes (dashed lines), considerably more pronounced discontinuities are observed when going from **9** to **10**, as the excitation energy shows a drastic decrease for all considered states.

As previously mentioned and evident in Figure 4, the first excitation energy of both neutral and cationic acenes decreases with increasing acene length. In case of the cations, this leads to the first excited doublet state increasingly mixing with the doublet ground state and the description using a single‐reference method, such as DFT (TD‐DFT), becomes increasingly poor. However, since the other state has the same multiplicity, the effect on the energy is small, which is why no drastic change in the excitation energies in Figure 4 is observed. This increasing degeneracy is however reflected in the SCF convergence issues encountered for acene cations larger than **9** 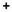 (see Section 2). In the case of neutral acenes, the closed‐shell singlet ground state also starts to mix with the lowest‐lying triplet state. Here a multi‐reference method is necessary for a proper theoretical treatment and therefore the quality of the description employing DFT decreases as the acenes get longer. In this case the effect on the energy is stronger compared to the cations, due to the mixing with the triplet state, which is observed in the strong change in excitation energies when going from **9** to **10** in Figure 4. While this decrease in the first excitation energy is a gradual process, TD‐DFT seems to reach a “breaking point” at **10** and **10** 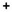 from where on the description suffers tremendously. The acene length where this happens will be dependent on the xc‐functional used.

Another aspect where this is reflected, is the orbital contributions to the transitions that make up the α‐ and β‐bands. In the smaller acenes **2**–**4** these are H→L+1 and H‐1→L, H→L+2 and H‐2→L for **5**–**7**, H→L+3 and H‐3→L for **8** and **9**. For **10**–**12** however the contributions change to again being H→L+1 and H‐1→L. In the cations on the other hand, the trend of the gap between the participating orbitals increasing is continued for **10**–**12**, which can be seen in Table S4.

## 6 Summary

The excited states of acene radical cations were thoroughly investigated and set into relation to their well‐known neutral counterparts, the 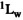, 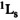 and 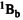 states.

A benchmark of the excitation energies of **2** 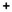, **3** 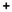 and **4** 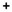 obtained using ADC(2), ADC(2)‐x, ADC(3) and IP‐ADC(3) against experiment was performed showing that ADC(3) and IP‐ADC(3) agree very well with experimental values, the MAPEs being 4.9%–7.3% and 2.5%–8.4% respectively. The results of the ADC(2) calculations were used for a more detailed analysis of the excited states and for a comparison to the 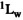, 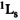, 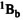 states of neutral acenes. By relation of analogous molecular orbitals of the neutral and cationic systems, it was observed that the characteristic states are still present, while naturally new states appear due to excitations into the SOMO. The cationic excited states that relate to the typical states of the neutral acenes by their orbital contributions were labeled 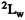, 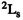 and 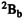.

In order to investigate larger acenes, a benchmark of the excitation energies obtained using the DFT functionals BLYP, B3LYP, BHHLYP and CAM‐B3LYP for **2** 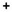
‐**5** 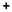 against experimental values was performed. The results show that BLYP shows a very good agreement with experiment for the smaller acenes. However, the difference in the performance of the functionals diminishes as the length of the acene increases.

The excited states of **2** 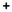
‐**12** 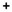 and **2**–**12** for comparison were calculated at the TDA/CAM‐B3LYP level of theory. As is expected, the excitation energies decrease with increasing acene length and further are converging towards a certain value. It is clearly observed that both the description of the neutral and cationic acenes suffer with increasing length, because their respective ground states gain more and more multi‐configurational character. The correct theoretical treatment of longer acenes therefore requires the use of multi‐reference methods, such as CASSCF, CASPT2, MRCI, and so forth. This is however almost entirely prohibited by their large size, which is why longer acenes are very challenging systems to study theoretically.

## Supporting information


**Data S1.** Supplementary Information.

## Data Availability

The data that support the findings of this study are available from the corresponding author upon reasonable request.
