# Supplementary material for: The Relation Between the Excited Electronic States of Acene Radical Cations and Neutrals—A Computational Analysis
Source: J Comput Chem. 2025 Apr 8;46(10):e70095. doi: 10.1002/jcc.70095 (PMC11976519; doi:10.1002/jcc.70095)
Supplement: Supplementary file 1 — Data S1. Supplementary Information. [file JCC-46-0-s001.pdf]

Supporting Information - The Relation between the Excited Electronic States of Acene Radical Cations and Neutrals - A Computational Analysis

Contents

1 Assignment of Cation HF Orbitals to Neutral HF Orbitals **1**

2 Simulated Absorption Spectra **3**

2.1 Simulated ADC Spectra of **2** and $\mathbf{2}^+$ 3

2.2 Simulated ADC Spectra of **3** and $\mathbf{3}^+$ 5

2.3 Simulated ADC Spectra of **4** and $\mathbf{4}^+$ 7

2.4 Simulated TDA spectra of $\mathbf{2}^+$-$\mathbf{12}^+$ 9

3 Orbital Contributions to TDA Cation Excited States **11**

1 Assignment of Cation HF Orbitals to Neutral HF Orbitals

α		neutral		β		neutral	
LUMO + 1	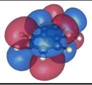	LUMO + 1	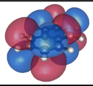	LUMO + 2	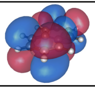	LUMO + 1	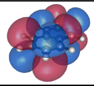
LUMO	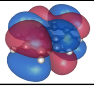	LUMO	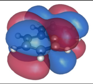	LUMO + 1	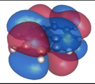	LUMO	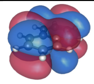
HOMO	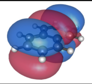	HOMO – 1	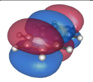	LUMO	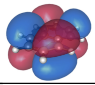	HOMO	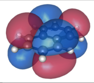
HOMO – 1	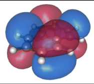	HOMO	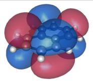	HOMO	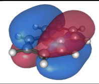	HOMO – 2	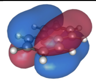

Figure S1: Example for the assignment of the orbitals obtained at HF/6-311G* of $\mathbf{2}^+$ to those of **2** according to their shape.

Table S1: Assigment of the orbitals obtained at HF/6-311G* of **2**$^+$ to those of **2** according to their shape and irreducible representation.

orbital no.	α-orbital	assignment neutral	β-orbital	assigment neutral
38	L+3	L+5	L+4	L+5
37	L+2	L+3	L+3	L+2
36	L+1	L+1	L+2	L+1
35	L	L	L+1	L
34	H	H-1	L	H
33	H-1	H	H	H-2
32	H-2	H-2	H-1	H-1
31	H-3	H-3	H-2	H-3

Table S2: Assigment of the orbitals obtained at HF/6-311G* of **3**$^+$ to those of **3** according to their shape and irreducible representation.

orbital no.	α-orbital	assignment neutral	β-orbital	assigment neutral
51	L+3	L+3	L+4	L+3
50	L+2	L+2	L+3	L+2
49	L+1	L+1	L+2	L+1
48	L	L	L+1	L
47	H	H	L	H
46	H-1	H-1	H	H-2
45	H-2	H-2	H-1	H-1
44	H-3	H-3	H-2	H-3

Table S3: Assigment of the orbitals obtained at HF/6-311G* of **4**$^+$ to those of **4** according to their shape and irreducible representation.

orbital no.	α-orbital	assignment neutral	β-orbital	assigment neutral
64	L+3	L+6	L+4	L+3
63	L+2	L+1	L+3	L+2
62	L+1	L+2	L+2	L+1
61	L	L	L+1	L
60	H	H	L	H
59	H-1	H-1	H	H-2
58	H-2	H-2	H-1	H-1
57	H-3	H-3	H-2	H-3

2 Simulated Absorption Spectra

2.1 Simulated ADC Spectra of 2 and 2^+

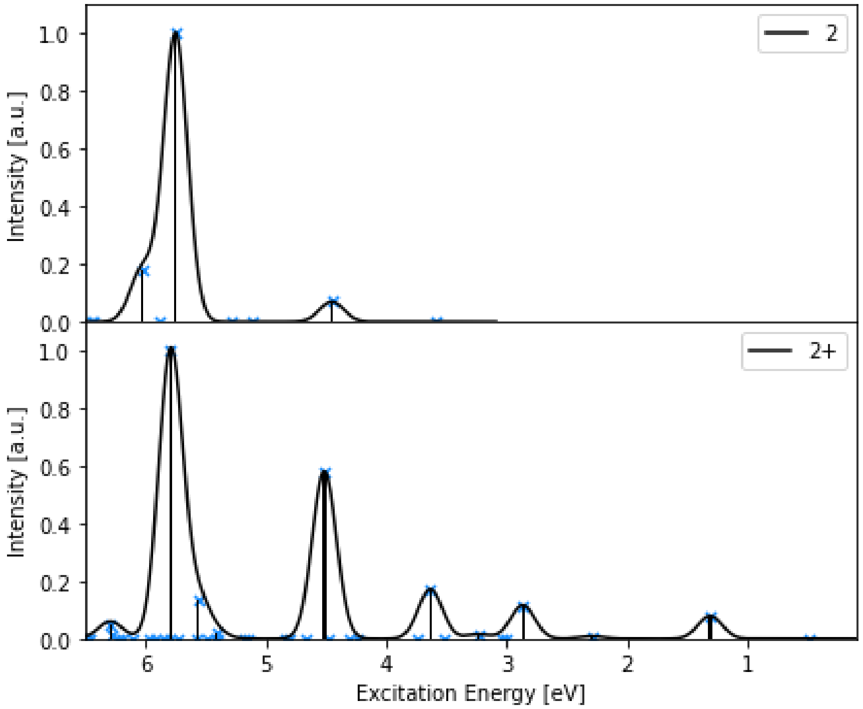

Figure S2: Simulated absorption spectra of **2** (top) and $\mathbf{2}^+$ (bottom) using excitation energies obtained with ADC(2)-x/6-311G*.

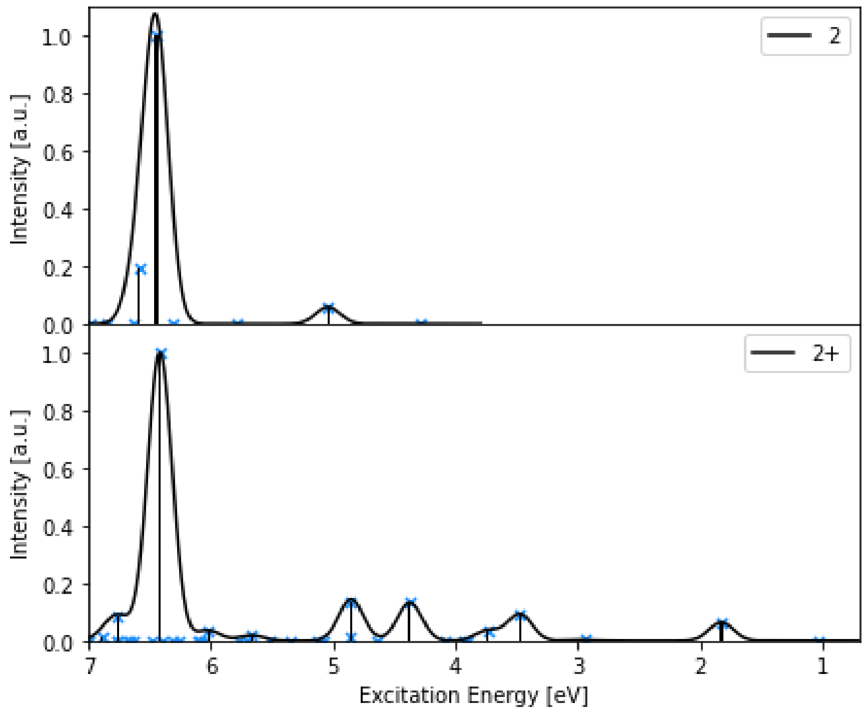

Figure S3: Simulated absorption spectra of **2** (top) and $\mathbf{2}^+$ (bottom) using excitation energies obtained with ADC(3)/6-311G*.

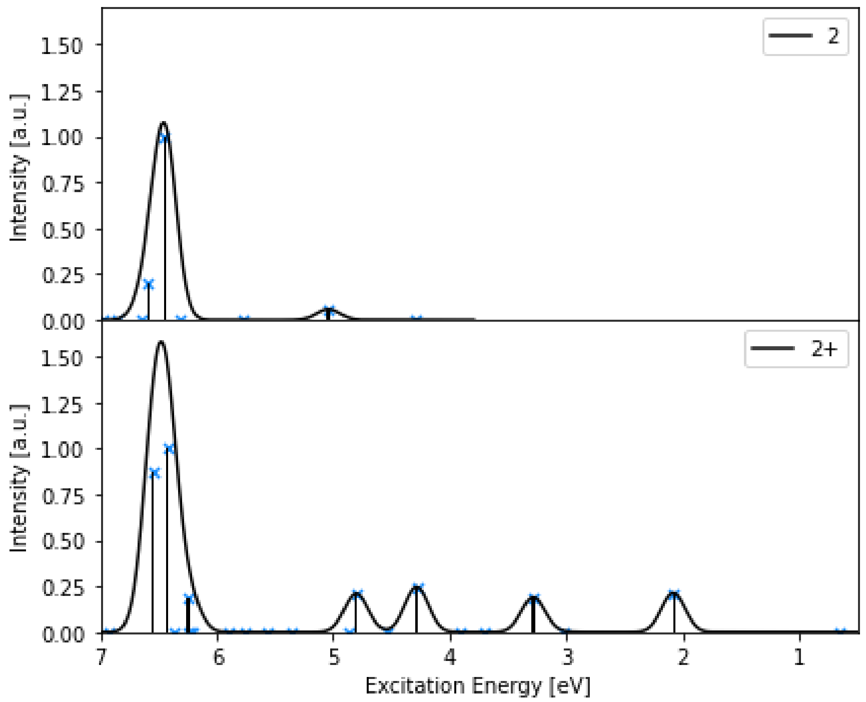

Figure S4: Simulated absorption spectra of **2** (top) and $\mathbf{2}^+$ (bottom) using excitation energies obtained with ADC(3) and IP-ADC(3)/6-311G*.

2.2 Simulated ADC Spectra of 3 and 3^+

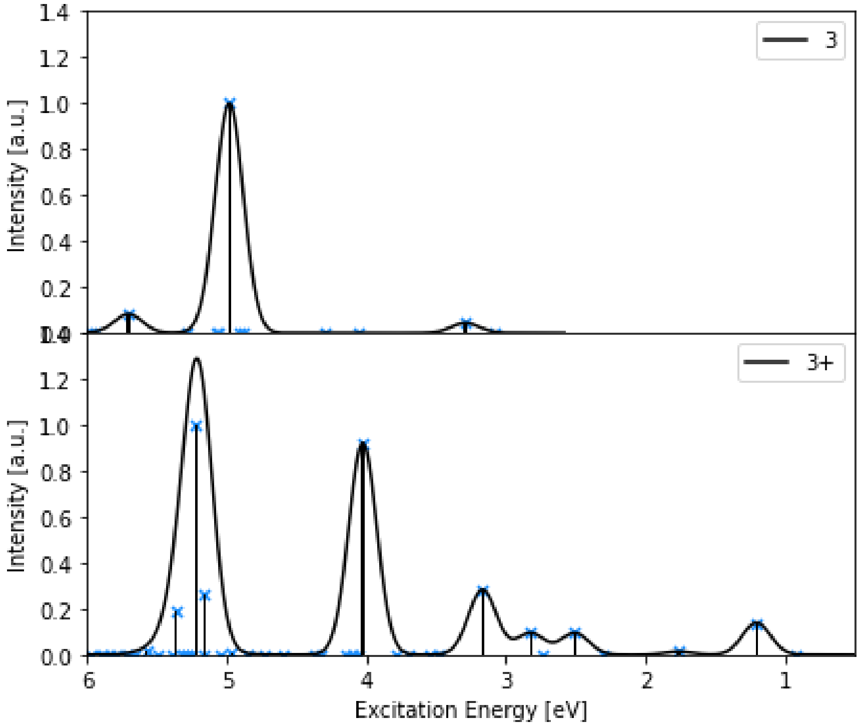

Figure S5: Simulated absorption spectra of **3** (top) and $\mathbf{3}^+$ (bottom) using excitation energies obtained with ADC(2)-x/6-311G*.

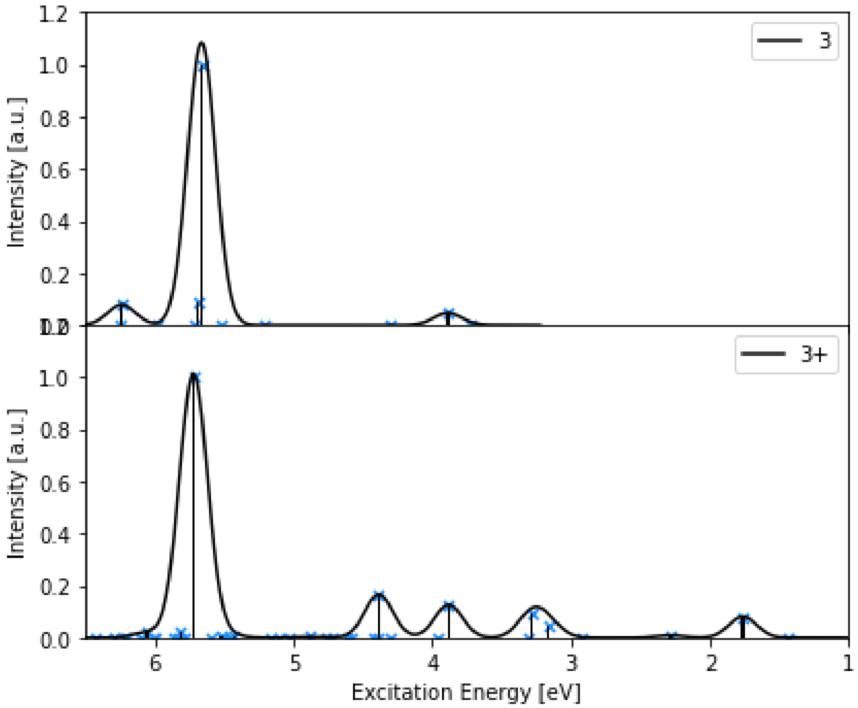

Figure S6: Simulated absorption spectra of **3** (top) and $\mathbf{3}^+$ (bottom) using excitation energies obtained with ADC(3)/6-311G*.

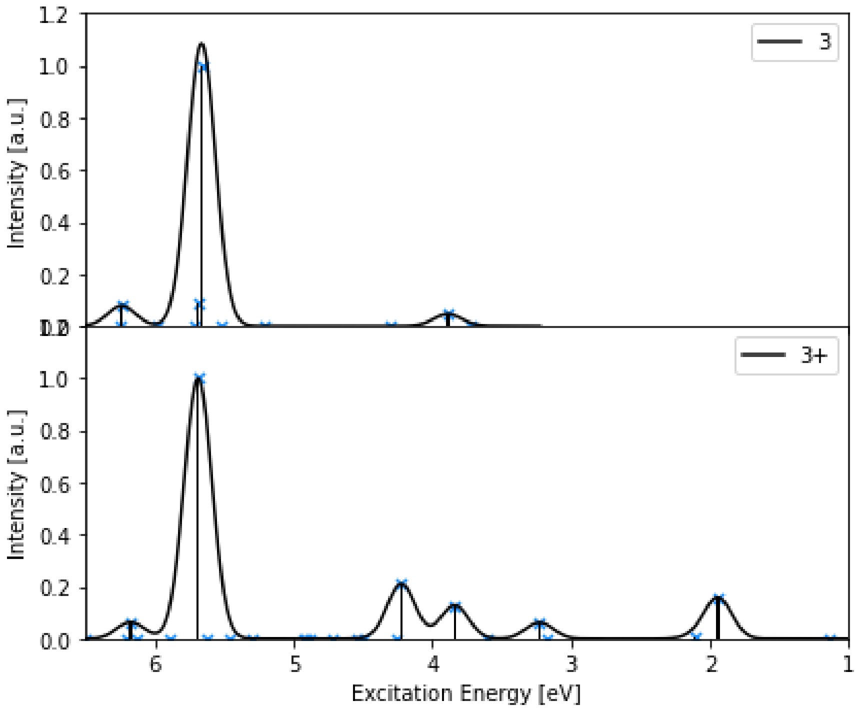

Figure S7: Simulated absorption spectra of **3** (top) and $\mathbf{3}^+$ (bottom) using excitation energies obtained with ADC(3) and IP-ADC(3)/6-311G*.

2.3 Simulated ADC Spectra of 4 and 4^+

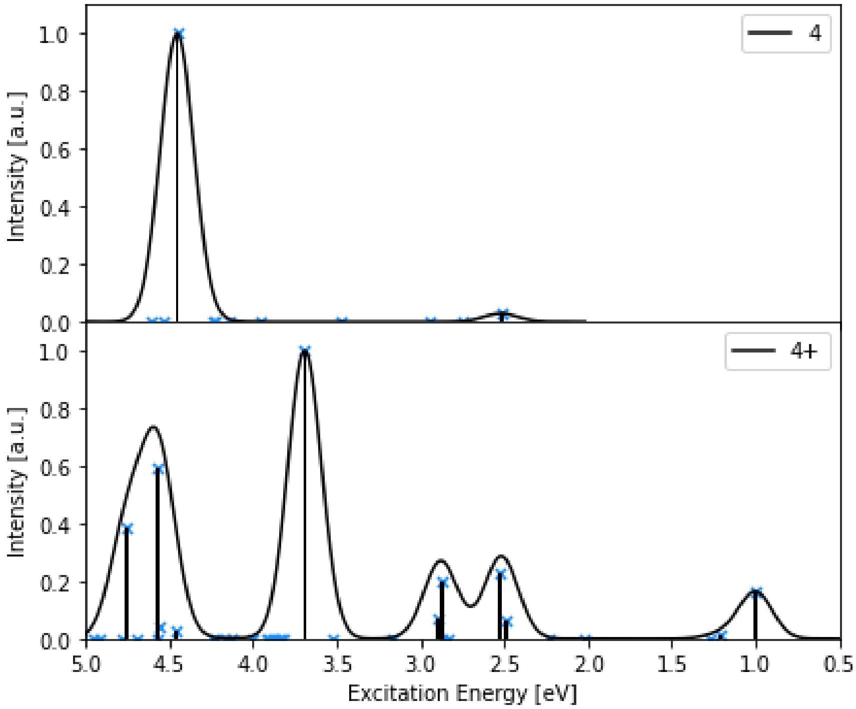

Figure S8: Simulated absorption spectra of **4** (top) and $\mathbf{4}^+$ (bottom) using excitation energies obtained with ADC(2)-x/6-311G*.

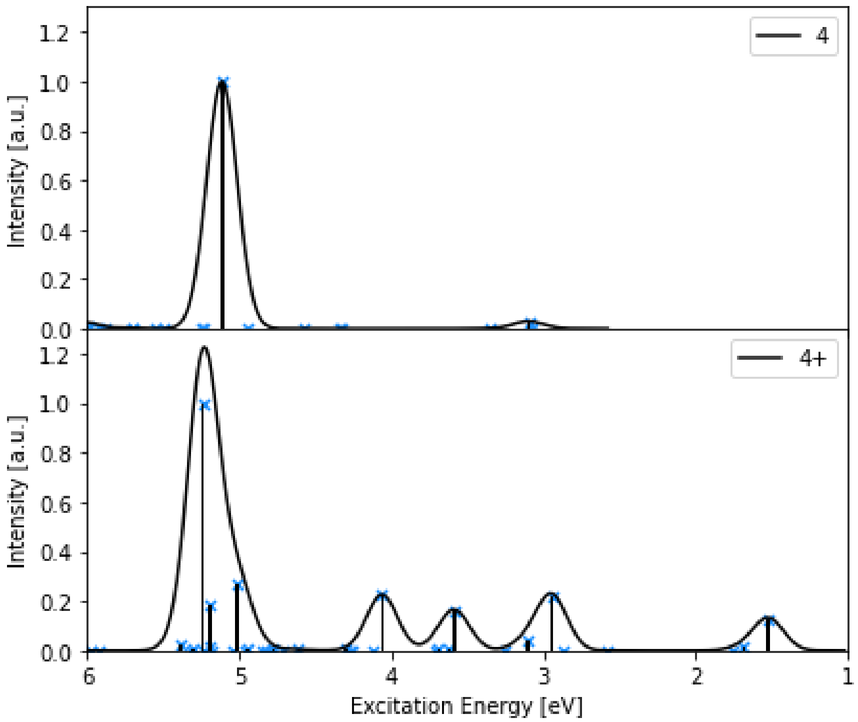

Figure S9: Simulated absorption spectra of **4** (top) and $\mathbf{4}^{+}$ (bottom) using excitation energies obtained with ADC(3)/6-311G*.

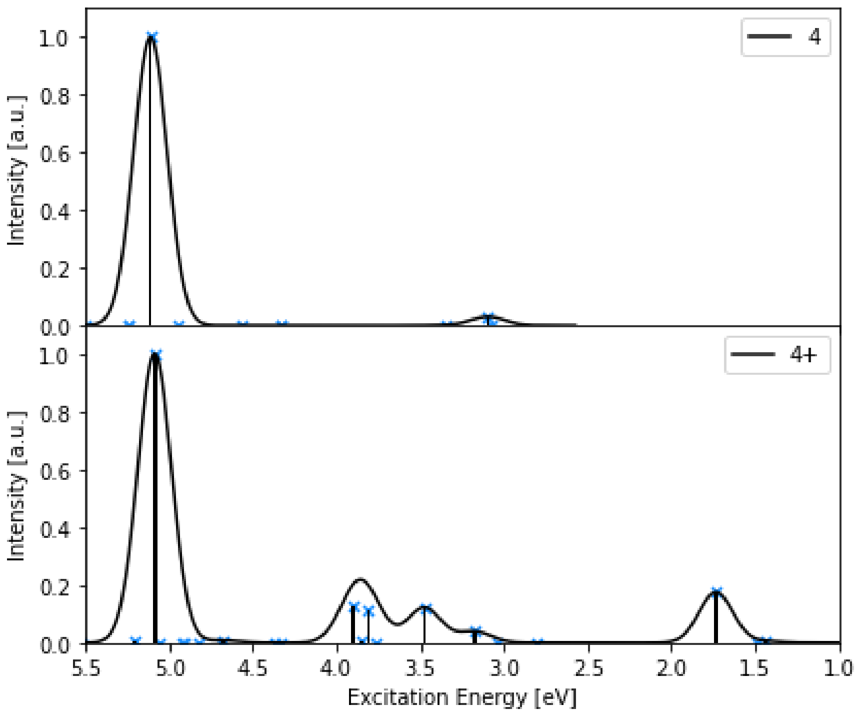

Figure S10: Simulated absorption spectra of **4** (top) and $\mathbf{4}^{+}$ (bottom) using excitation energies obtained with ADC(3) and IP-ADC(3)/6-311G*.

2.4 Simulated TDA spectra of 2^+-12^+

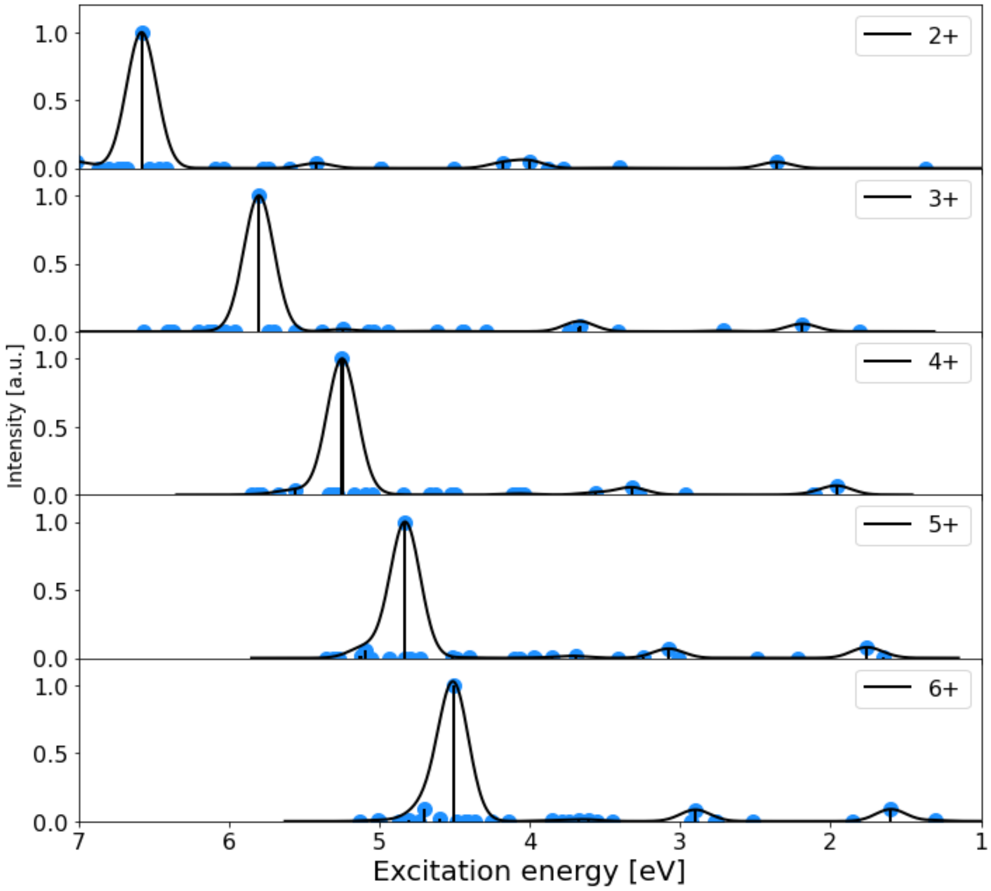

Figure S11: Simulated absorption spectra of $\mathbf{2}^+$-$\mathbf{6}^+$ using excitation energies obtained with TDA/CAM-B3LYP/6-311G*.

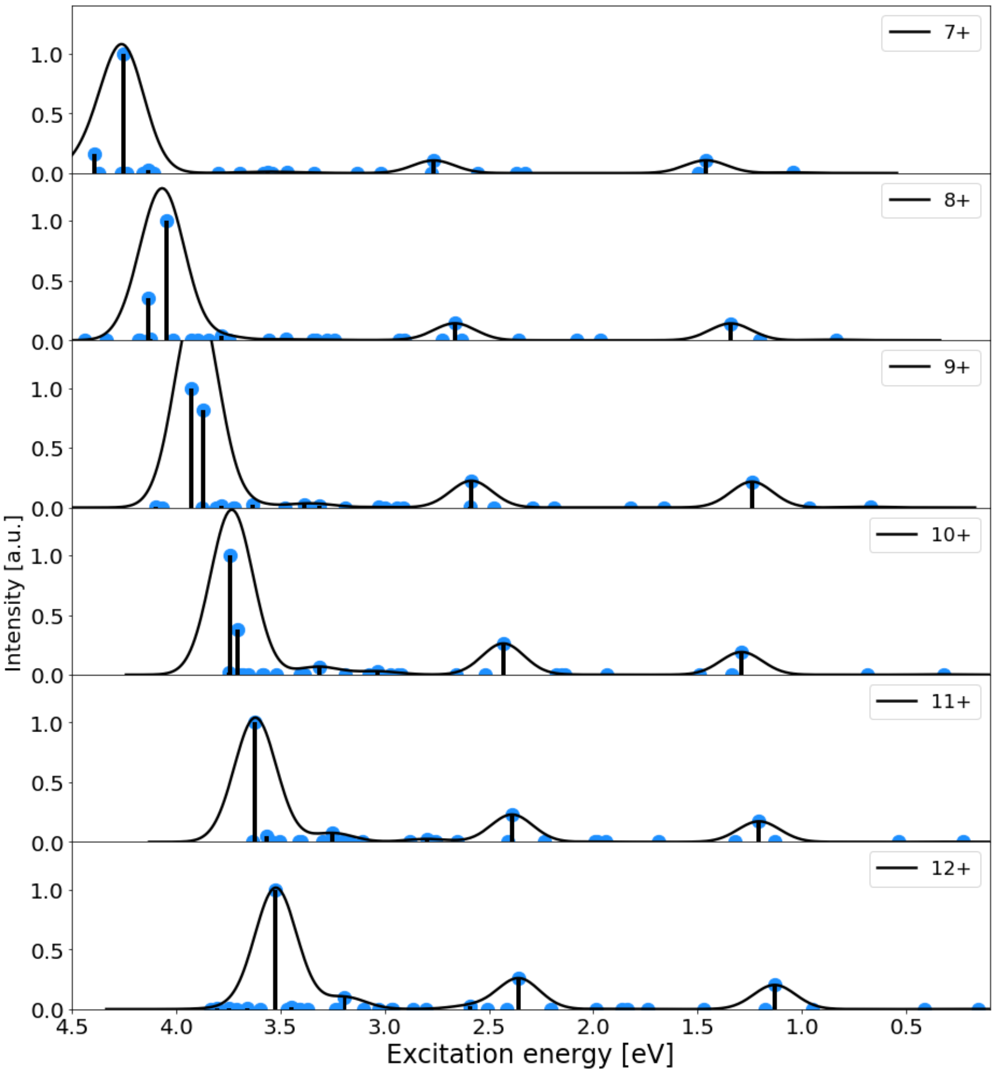

Figure S12: Simulated absorption spectra of $\mathbf{7}^{+}$-$\mathbf{12}^{+}$ using excitation energies obtained with TDA/CAM-B3LYP/6-311G*.

3 Orbital Contributions to TDA Cation Excited States

Table S4: Evolution of contributions to α- and β-states of neutral and cationic acenes.

Molecule	α- and β-band	cation α-band	cation β-band
2	H-1 $\rightarrow$ L	αH $\rightarrow$ αL+1	αH $\rightarrow$ αL+1
	H $\rightarrow$ L+1	βH $\rightarrow$ βL+1	βH $\rightarrow$ βL+1
3	H-1 $\rightarrow$ L	αH-1 $\rightarrow$ αL	αH $\rightarrow$ αL+1
	H $\rightarrow$ L+1	βH-1 $\rightarrow$ βL+1	βH-1 $\rightarrow$ βL+1
4	H-1 $\rightarrow$ L	αH-1 $\rightarrow$ αL	αH $\rightarrow$ αL+2
	H $\rightarrow$ L+1	βH-1 $\rightarrow$ βL+1	βH-1 $\rightarrow$ βL
5	H-2 $\rightarrow$ L	αH-2 $\rightarrow$ αL	αH $\rightarrow$ αL+2
	H $\rightarrow$ L+2	βH-1 $\rightarrow$ βL+1	βH-1 $\rightarrow$ βL+1
6	H-2 $\rightarrow$ L	αH-2 $\rightarrow$ αL	αH $\rightarrow$ αL+2
	H $\rightarrow$ L+2	βH-2 $\rightarrow$ βL+1	βH-2 $\rightarrow$ βL+1
7	H-2 $\rightarrow$ L	αH-2 $\rightarrow$ αL	αH $\rightarrow$ αL+2
	H $\rightarrow$ L+2	-	βH-2 $\rightarrow$ βL+1
8	H-3 $\rightarrow$ L	αH-3 $\rightarrow$ αL	αH $\rightarrow$ αL+3
	H $\rightarrow$ L+3	-	βH-2 $\rightarrow$ βL+1
9	H-3 $\rightarrow$ L	αH-3 $\rightarrow$ αL	αH $\rightarrow$ αL+3
	H $\rightarrow$ L+3	-	βH-3 $\rightarrow$ βL+1
10	H-1 $\rightarrow$ L	αH $\rightarrow$ αL+3	αH $\rightarrow$ αL+3
	H $\rightarrow$ L+1	βH-3 $\rightarrow$ βL+1	-
11	H-1 $\rightarrow$ L	αH $\rightarrow$ αL+4	αH $\rightarrow$ αL+4
	H $\rightarrow$ L+1	βH-3 $\rightarrow$ βL+1	-
12	H-1 $\rightarrow$ L	αH $\rightarrow$ αL+4	αH $\rightarrow$ αL+4
	H $\rightarrow$ L+1	βH-4 $\rightarrow$ βL+1	-
